# CYP1B1-AS1 regulates CYP1B1 to promote *Coxiella burnetii* pathogenesis by inhibiting ROS and host cell death

**DOI:** 10.21203/rs.3.rs-5390645/v1

**Published:** 2024-12-11

**Authors:** Aryashree Arunima, Seyednami Niyakan, Samantha M. Butler, Sabrina D. Clark, Anna Pinson, Doyoung Kwak, Xiaoning Qian, Paul de Figueiredo, Erin J. van Schaik, James E. Samuel

**Affiliations:** 1 Department of Microbial Pathogenesis and Immunology, Texas A&M University Health Science Center, Bryan, TX 77807, USA.; 2 Department of Electrical and Computer Engineering, Texas A&M University, College Station, TX 77843, USA.; 3 Bond Life Sciences Center, University of Missouri, Columbia, MO 65211, USA.; 4 Department of Molecular Microbiology and Immunology, University of Missouri School of Medicine, Columbia, MO 65212, USA.; 5 Department of Veterinary Pathobiology, University of Missouri, Columbia, MO 65211, USA.

**Keywords:** *Coxiella burnetii*, CYP1B1-AS1, lncRNA marker, CYP1B1, reactive oxygen species (ROS), apoptosis, inflammation, bacterial pathogenesis, mitochondrial effector, CBU_0937

## Abstract

*Coxiella burnetii* (Cb), the causative agent of Q fever, replicates within host macrophages by modulating immune responses through poorly understood mechanisms. Long non-coding RNAs (lncRNAs) are emerging as critical regulators of inflammation, yet their role in Cb pathogenesis remains largely unexplored. Here, we employed a global transcriptomic approach to identify lncRNAs specific to Cb infection in THP-1 derived macrophages, compared to 15 other microbial infections. CYP1B1-AS1 was uniquely regulated in a spatio-temporal manner during Cb infection. Promoter assays revealed that CYP1B1-AS1 is transcribed by AHR from a bidirectional promoter, enhancing CYP1B1 expression in *cis*. Inhibition of CYP1B1-AS1 and CYP1B1 increased reactive oxygen species (ROS), mitochondrial membrane depolarization, and apoptosis, suggesting their role in dampening host cell death. Additionally, immunoprecipitation followed by mass spectrometry identified the mitochondrially localized Cb effector CBU_0937 as an interactor of the CYP1B1 enzyme. These events facilitate Cb intracellular survival. Our findings identify CYP1B1-AS1 as a potential molecular target for combating Cb infection.

## Introduction

Infectious diseases remain a leading cause of mortality worldwide, with the World Health Organization (WHO) reporting approximately 17 million cases annually, and around 50,000 deaths related to chronic inflammation from diseases such as typhoid^1^, brucellosis^2^, tuberculosis^3, 4^, and gastroenteritis^5^. The rise of antibiotic resistance and the prevalence of lifestyle-associated chronic inflammation further complicate the treatment of these conditions^1, 6^. Therefore, understanding the underlying mechanisms and identifying novel disease regulators are critical for developing novel diagnostics and therapies.

*Coxiella burnetii* (Cb), the causative agent of Q fever, is an obligate intracellular Gram-negative pathogen. It primarily infects monocytes and macrophages, in a replicative niche called as *Coxiella*-containing vacuoles (CCVs)^7^. The bacterium utilizes a type IVB secretion system (T4SS) to translocate a variety of effectors into host cells, modulating immune responses such as apoptosis and inflammasomes to promote endocytic trafficking and intracellular replication^8, 9, 10^ Despite extensive studies, how *Coxiella* evades innate immune responses and establishes chronic infection remains poorly understood.

The Nine Mile strain of Cb exists in two variants: phase I (NMI), with full-length lipopolysaccharides (LPS), and phase II (NMII), which has truncated LPS^11^. While infection is usually transmitted via inhalation of contaminated aerosols or dust, resulting in an acute, self-limiting flu-like illness, chronic cases can lead to severe complications such as endocarditis and lung fibrosis^12^. Despite its global incidence, there is currently no clinically approved vaccine in the United States, and the only licensed vaccine, Q-Vax, is restricted to Australia due to adverse effects in previously exposed populations^13^. The standard treatment for chronic Q fever involves long-term antibiotic therapy^14^, underscoring the urgent need for novel therapeutic and diagnostic approaches.

Long non-coding RNAs (lncRNAs), a class of regulatory transcripts over 200 nucleotides in length, have recently emerged as important regulators of inflammation and immune responses^15, 16^. Dysregulation of lncRNAs has been implicated in various diseases, and they are increasingly recognized as potential diagnostic and prognostic biomarkers^17^. For example, the lncRNA NEAT1 is involved in macrophage function and is linked to diseases such as cancer, neurodegenerative disorders, and infectious diseases like *Salmonella*^18^ and *Mycobacterium tuberculosis* infections^19^. However, the role of lncRNAs in Cb infection remains largely unexplored.

Cb offers a unique model for studying intracellular replication due to its reliance on the lysosome-matured CCVs for survival, a niche that is non-permissive for most intracellular pathogens^7^. In this study, we sought to identify lncRNAs uniquely expressed during Cb infection and their potential regulatory roles in intracellular replication and immune response modulation. Our central hypothesis is that the distinct lncRNA signature associated with Cb infection plays a critical role in controlling pathogen replication and host immune responses.

To test this, we conducted a comprehensive RNA sequencing (RNA-seq) analysis to identify differentially expressed (DE)-lncRNAs in macrophages infected with Cb compared to those infected with a panel of pathogenic and non-pathogenic bacteria (Table 1). THP-1 derived macrophages were used as model to screen human-specific lncRNAs associated with Cb infection. Among the identified candidates, CYP1B1-AS1 was identified as a key lncRNA, uniquely upregulated during Cb infection.

Our *in vitro* studies demonstrated that CYP1B1-AS1 regulates the expression of its target gene, CYP1B1, a mitochondrial enzyme involved in reactive oxygen species (ROS) production and mitochondrial function^20, 21^. Regulatory region prediction analysis identified aryl hydrocarbon receptor (AHR) as a potential transcription factor (TF), and agonist assays further confirmed that AHR activation induces the expression of both CYP1B1-AS1 and CYP1B1. Dysregulation of CYP1B1-AS1 and CYP1B1 led to impaired mitochondrial homeostasis, elevated ROS production, and apoptosis, which likely contributed to the observed reduction in Cb counts within host cells. Silencing CYP1B1-AS1 notably decreased Cb replication and triggered an inflammatory response, suggesting its involvement in modulating host-pathogen interactions. We also identified CBU_0937, a mitochondrial-localized *Coxiella* protein, as an interactor of CYP1B1, potentially playing a key role in regulating host mitochondrial functions. Importantly, prior to this study, the role of Cb in regulating mitochondrial function and AHR signaling was not well understood. Our findings provide the first evidence linking Cb to these critical cellular processes.

Overall, these findings highlight CYP1B1-AS1 as a crucial regulator of Cb pathogenesis. By identifying its role in inflammation, ROS production, and cell death, our study sheds light on a novel immune signaling pathway that could be exploited for the development of RNA-based therapeutics targeting Q fever.

## Results

### Identification of Cb infection-associated lncRNAs

Defining early-phase transcriptomic changes in lncRNAs is essential to understand their roles as markers and regulators of immune responses. To identify potential lncRNA markers for Cb infection, we compared global RNA-seq data from phorbol 12-myristiate-12 acetate (PMA)-treated THP-1 cells (THP-1 macrophages) infected with various bacterial strains for 1 h (Table S1). The workflow for sample preparation, RNA sequencing, and data analysis is outlined in Fig. S1a and S1b. We analyzed in total 16 bacterial strains, including three Cb strains (virulent and attenuated) and 13 other strains with varying pathogenic potentials (pathogen, attenuated pathogen, opportunistic pathogen, and non-pathogen control). DE transcripts during Cb infection were analyzed to identify uniquely expressed lncRNAs, comparing them to attenuated strains of *C. burnetii* Nine Mile Phase II dotA::Tn (CbA or Δ*dotA*), *C. burnetii* Nine Mile Phase II dotB::Tn (CbB), Enterohemorrhagic *Escherichia coli* O157 (EcT), Enterohemorrhagic *Escherichia coli* O157 Δstx (nontoxigenic) (EcN), *Francisella novicida* U112 (Fn), *Pseudomonas aeruginosa* PAO1 (Pa), *Staphylococcus aureus* JE2 (Sa), *Salmonella enterica* subsp. *Typhimurium* SL1344 (STm), *Rhizobium radiobacter* (Rr), *Micrococcus luteus* (Ml), *Enterococcus faecalis* (Ef), *Brucella melitensis* Δ*vjbR* (Bmv), and three non-pathogenic controls: *Escherichia coli* DH5α (Ec5), *Listeria innocua* (Li), and *Bacillus subtilis* P31K6 (Bs). This diverse selection enabled us to identify fundamental cellular responses to different bacterial infections, independent of colonization sites, therapeutic regimens, or co-infections.

**Fig 1.**
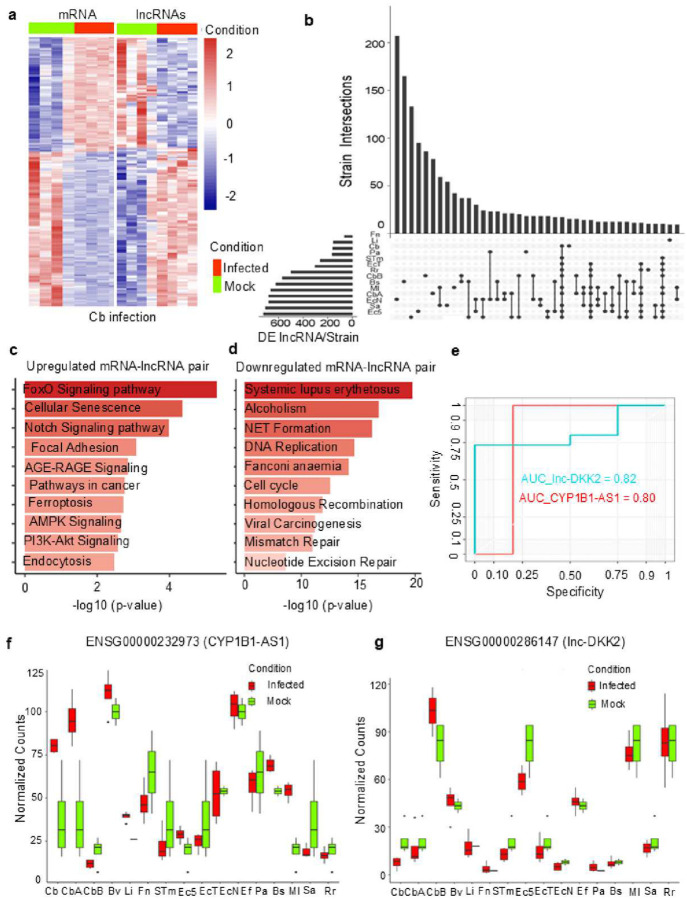
CYP1B1-AS1 and lnc-DKK2 are uniquely regulated in *Coxiella burnetii* (Cb) infection. (a) Hierarchical clustering heatmap of differentially expressed (DE) mRNAs and DE lncRNAs. Each row represents a gene, and columns represent sample replicates. Relative expression levels are shown on a color scale, with red indicating upregulation and blue indicating downregulation. Fold changes are presented as normalized RPKM (Reads Per Kilobase of transcript per Million mapped reads) and log2-transformed. (b) Upset plot showing overlaps in DE lncRNAs among various strains: *C. burnetii* Nine Mile Phase II (Cb), *C. burnetii* Nine Mile Phase II *dotA*::Tn (CbA), *C. burnetii* Nine Mile Phase II *dotB*::Tn (CbB), *Escherichia coli* DH5α (Ec5), *Enterohemorrhagic E. coli* O157 (EcT), *Enterohemorrhagic E. coli* O157Δstx (EcN), *Bacillus subtilis* P31K6 (Bs), *Francisella novicida* U112 (Fn), *Pseudomonas aeruginosa* PAO1 (Pa), *Staphylococcus aureus* JE2 (Sa), *Salmonella enterica* subsp. Typhimurium SL1344 (STm), *Rhizobium radiobacter* (Rr), *Micrococcus luteus* (Ml), *Listeria innocua* (Li), *Enterococcus faecalis* (Ef), and *Brucella melitensis* Δ*vjbR* (Bmv). Bars with single dots represent the number of unique DE lncRNAs for each strain. The bar plot on the lower left shows the total number of DE lncRNAs for each strain. KEGG pathway analysis of (c) upregulated mRNAs associated with DE lncRNAs and (d) downregulated mRNAs associated with DE lncRNAs, with color gradient indicating the proportion of transcripts for each annotation. The top 10 significantly enriched terms are shown, filtered at P < 0.05. (e) Receiver Operating Characteristic (ROC) curve analysis displaying the diagnostic value of lnc-DKK2 and CYP1B1-AS1 in different infection models, with Area Under the Curve (AUC) values at P < 0.01 and 95% confidence intervals (CI). (f) Boxplot validating the expression of CYP1B1-AS1 and (g) lnc-DKK2 across infection models, with fold changes represented as normalized RPKM counts.

Global analysis revealed several DE-lncRNAs (Data S1), with a focus on *cis*-acting lncRNAs located in proximity to DE-mRNAs (Data S2) to investigate likely role in regulation of immune responses. Our RNA-seq dataset consisted of 64 samples, with alignment properties and raw counts provided in Data S3. Principal component analysis (PCA) (Fig. S1c) showed 33.98% variance between uninfected (Mock) macrophages and those infected with different bacteria, with 14.42% variance across infected macrophages. The scatter plots showing differential regulation of lncRNA and mRNA are shown in Fig. S1d and Fig. S2 (a-m). Using a log_2_ fold-change (FC) cut-off) of ≥ 1 or ≤ −1 at P < 0.05, we identified 1692 DE genes in Cb-infected macrophages, with 748 upregulated and 944 downregulated (Data S4a). Hierarchical clustering analysis revealed 1531 DE-mRNAs and 161 DE-lncRNAs (Fig. 1a, Data S4a). The majority of DE-lncRNAs were intergenic or anti-sense, with some classified as novel transcripts (Data S4b). LncRNA for potential *cis*- and *trans*-regulation of mRNA were predicted based on their genomic proximity, synteny, and sequence similarity^22, 23^. We identified 55 DE-lncRNA-mRNA pairs based on proximity, synteny, and sequence similarity (Data S4c).

Next, we sought to identify Cb-specific lncRNAs by comparing DE-lncRNAs from other infections and non-pathogenic controls. Our upset plot analysis identified two DE-lncRNAs; ENSG00000232973 (CYP1B1-AS1) and ENSG00000286147 (lnc-DKK2), were unique to Cbinfected macrophages (Fig. 1b). CYP1B1-AS1 was upregulated in both Cb and Cb NMII *dotA*::Tn (CbA) infections, while lnc-DKK2 was specifically downregulated in Cb infection.

Genes in close spatial proximity are often functionally related and tend to exhibit correlated expression patterns^23, 24, 25^. To further understand these relationships, we performed a global functional enrichment analysis of all DE-lncRNAs based on their associated DE-mRNAs, annotating them according to their predicted functions. An overview of the enriched categories for transcriptome changes in response to Cb infection according to GO analysis is shown in Fig. S2 (n-s). The results revealed that mRNAs associated with upregulated lncRNAs were predominantly enriched in cytoplasmic vesicles and endocytic membranes (Fig. S2n), whereas downregulated genes were largely associated with nuclear compartments (Fig. S2q). Further GO-molecular function analysis showed that upregulated lncRNAs were enriched for activities such as NADPH oxidase regulation, which is involved in superoxide generation, and serine/threonine kinase activity (Fig. S2p). In contrast, the downregulated genes were primarily associated with DNA-binding functions (Fig. S2s). The top 10 functional annotations for up- and downregulated mRNAs, categorized by Kyoto Encyclopedia of Genes and Genomes (KEGG) pathways, are displayed in Fig. 1c and 1d respectively. KEGG pathway analysis highlighted that upregulated lncRNAs were involved in key signaling pathways like FOXO signaling, PI3K-Akt signaling, Notch signaling, and ferroptosis. Conversely, downregulated genes were enriched in pathways related to neutrophil extracellular trap (NET) formation, DNA repair, and other related processes.

CYP1B1 is a mitochondrial-localized, cytochrome P450 subfamily B member 1 enzyme with monooxygenase activity. It regulates reactive oxygen species (ROS) production and mitochondrial function^20, 21^. CYP1B1-AS1 is located in *cis* to CYP1B1 was predicted to regulate these functions based on its genomic proximity to the protein coding gene, while lnc-DKK2 was implicated in the regulation of the Wnt signaling pathway due to its association with DKK2^26^. Receiver-operating characteristic (ROC) curve analysis showed an area under the curve (AUC) of 0.80 for CYP1B1-AS1 and 0.82 for lnc-DKK2 at a 95% confidence interval (CI), P < 0.01 (Fig. 1e), indicating high sensitivity and specificity for detecting these lncRNAs as diagnostic markers. Furthermore, box plot analyses of both lncRNA signatures across different infections [Fig. 1(f–g)] showed that their expression trends, validated through quantitative real-time PCR (qPCR), correlated with RNA-seq results. Neither CYP1B1-AS1 nor lnc-DKK2 displayed significant expression changes in other infection models when compared to Mock controls (P ≥ 0.05) [Fig. S3 (a-m)]. However, in Cb-infected macrophages, CYP1B1-AS1 was approx. 7.94-fold upregulated (Fig. S3n), and lnc-DKK2 was approx. 33.3-fold downregulated (Fig. S3p).

In conclusion, our study employed a global comparative multi-infection model approach, which was instrumental in identifying CYP1B1-AS1 and lnc-DKK2 as potential lncRNA signatures specific to Cb infection. This multi-infection framework allowed us to distinguish these lncRNAs from those associated with other bacterial infections, emphasizing their unique roles in the host immune response to Cb pathogenesis.

### Spatio-temporal analysis and co-expression analysis of CYP1B1-AS1 and lnc-DKK2 during Cb infection

To further investigate the regulatory roles of these DE-lncRNAs, we analyzed their co-expression with associated protein-coding genes over time post-infection (p.i.) using qPCR. The qPCR data showed that CYP1B1 was approx. 7.66-fold upregulated, which correlated well with expression trends of CYP1B1-AS1 at 1 h p.i. [Fig. S3 (n-o)]. However, although DKK2 was upregulated by 3.01-fold, it did not correlate with the expression trends of lnc-DKK2 at 1 h p.i. [Fig. S3 (p-q)]. This prompted further investigation into the temporal co-expression patterns of these lncRNAs and their associated mRNAs during infection, both *in vitro* and *in vivo*.

**Fig 2.**
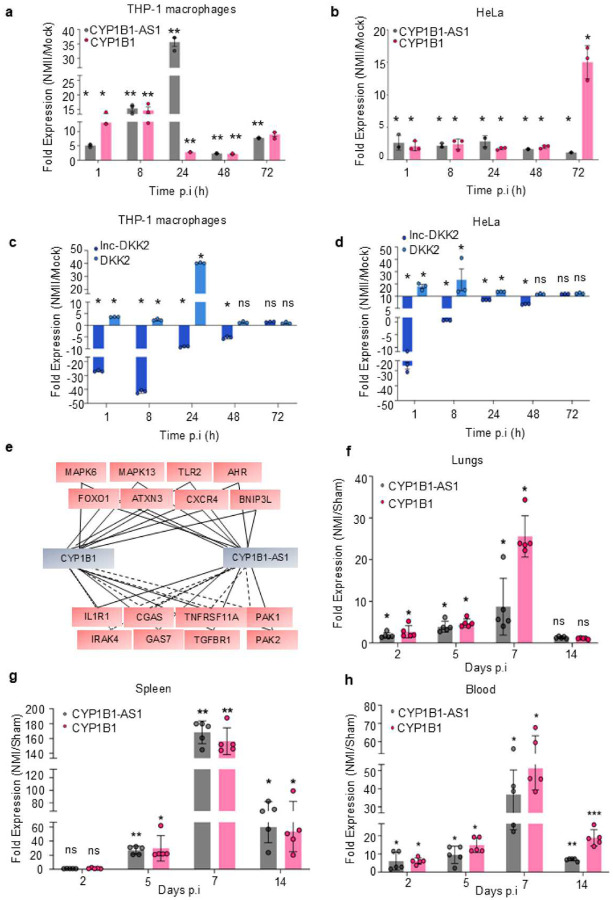
CYP1B1-AS1 and lnc-DKK2 are spatio-temporally regulated during Cb infection. Cells without siRNA treatment and infected with Cb were labelled as NMII. RNA was harvested at indicated time points for quantitative real-time PCR (qPCR) analysis. Gene expression was normalized against the endogenous control ACTB and compared to uninfected control (Mock). (a-b) Expression levels of CYP1B1-AS1 and CYP1B1 in: (a) THP-1 macrophages, and (b) HeLa cells. (c-d) Expression of lnc-DKK2 and DKK2 in (c) THP-1 macrophages, and (d) HeLa cells. Partial map of coding-noncoding (CNC) co-expression network analysis of (e) CYP1B1 and CYP1B1-AS1. The solid line between nodes represents a positive correlation, and dotted lines represents a negative correlation. Pearson correlation coefficient threshold: −0.9 to 0.9. (f-h) Lungs, spleen and blood harvested from mice groups, intra-tracheally infected with PBS (sham) or Cb Nine Mile I (NMI) at a dosage of 1×10^7^ genome equivalents (GE)/ml. RNA was isolated from homogenized tissues at the indicated days post-infection (p.i.) for qPCR analysis. Gene expression in NMI groups was normalized to ACTB and compared to the sham groups. Expression of CYP1B1-AS1 and CYP1B1 in (f) Lungs, (g) Spleen, and (h) Blood. For panels, (a-d), n=3; and for panels (f-h), n=5; error bars indicate mean ± SD. Statistical significance: *, P < 0.05; **, P < 0.01; ***, P < 0.0001; ****, P < 0.0001; ns, not significant, P ≥ 0.05; one-way ANOVA.

CYP1B1-AS1 and CYP1B1 exhibited similar expression patterns, being consistently upregulated from 1 to 72 h p.i. in THP-1 macrophages (Fig. 2a), HeLa cells (Fig. 2b), and HEK293T cells (data not shown). In contrast, lnc-DKK2 was significantly downregulated between 1 and 48 h p.i., while DKK2 was upregulated until 24 h p.i. [Fig. 2(c–d)]. This suggests that lnc-DKK2 and DKK2 do not exhibit *cis* co-expression, indicating that lnc-DKK2 may regulate a different target *in trans*.

We extended our analysis to four model pathogens: *Legionella pneumophila* (Lp; *L. pneumophila*) (evolutionarily related to Cb)^11^, Bmv, Li, and STm. While lnc-DKK2 showed increased expression at 48 h p.i., there was no significant correlation with DKK2 expression cells infected with these strains [Fig. S4 (a-h)]. Additionally, CYP1B1 was upregulated at various time points in these infections, suggesting a potentially alternative regulatory mechanism by these pathogens [Fig. S4 (m-p)]. Importantly, CYP1B1-AS1 expression remained unchanged in these infections, confirming its specificity as a marker for Cb infection [Fig. S4 (i-l)].

Although several lncRNAs, such as NEAT1, lincRNA-Cox2, and lincRNA-EPS, are known to regulate inflammasomes and gene expressions during bacterial or viral infections^16, 17^, their role as immune checkpoint regulators or diagnostic biomarkers in severe inflammatory infectious diseases is still emerging^17^. Our next objective was to assess the clinical relevance of CYP1B1-AS1 and lnc-DKK2 and explore their correlation with other DE genes in the Cb-infected transcriptome. Given the lack of high-throughput platforms annotated with reliable clinical outcomes for Q-fever or other infections, we performed a coding-noncoding (CNC) network analysis using the “guilt-by-association” approach^27^ to investigate the clinical relevance of DE genes. This analysis identified a set of lncRNAs and mRNAs most strongly correlated with CYP1B1-AS1, CYP1B1, lnc-DKK2, and DKK2 [Fig. S5 (a-b); Data S5]. Functional enrichment analysis of these correlated genes, based on GO and KEGG pathways, is shown in Fig. S5(c-f).

A partial map of the CNC network for CYP1B1-AS1 and CYP1B1 is shown in Fig. 2e. An interesting outcome of this analysis was the correlation of AHR with CYP1B1-AS1 and CYP1B1. AHR signaling is known to be induced in various respiratory viral^28^ and enteric bacterial infections^29^ and is a well-established transcriptional activator of the CYP1B1 enzyme^30^. In contrast, lnc-DKK2 and DKK2 showed weaker associations with DE genes in the Cb-infected transcriptome (Fig. S6a, Data S5), indicating that the transcriptome correlations predominantly centered on the CYP1B1-AS1/CYP1B1 network.

Next, we conducted a spatio-temporal co-expression study of CYP1B1-AS1 and CYP1B1 in mice infected with the Cb-NMI strain. A schematic of the workflow is provided in Fig. S6b. The infected mice were monitored for clinical signs of illness, such as weight loss (Fig. S6c) and splenomegaly (Fig. S6d), before proceeding with transcript analysis from blood, spleen, and lung tissues. Based on synteny conservation and the positional relationship between the two human genes, we identified the mouse orthologs CYP1B1 (ENSMUSG00000024087) and the associated anti-sense lncRNA Gm33055 (ENSMUSG00000117426), both sharing a common regulatory region upstream of the transcription start site (TSS; +1). Although sequence conservation between human CYP1B1-AS1 and mouse Gm33055 was low, we considered Gm33055 a candidate ortholog of CYP1B1-AS1 in this study. While further research is needed to fully characterize this lncRNA, it is notable that both CYP1B1 and the lncRNA demonstrated a *cis*-dependent co-expression pattern, consistent with our previous findings [Fig. 2(f–h)].

These findings highlight the regulatory importance of CYP1B1-AS1 in driving the co-expression of CYP1B1, identifying these genes as unique targets for further study into their mechanistic role during Cb infection.

### CYP1B1-AS1 is transcribed from a shared bidirectional promoter and regulates CYP1B1 *in* *cis*

**Fig 3.**
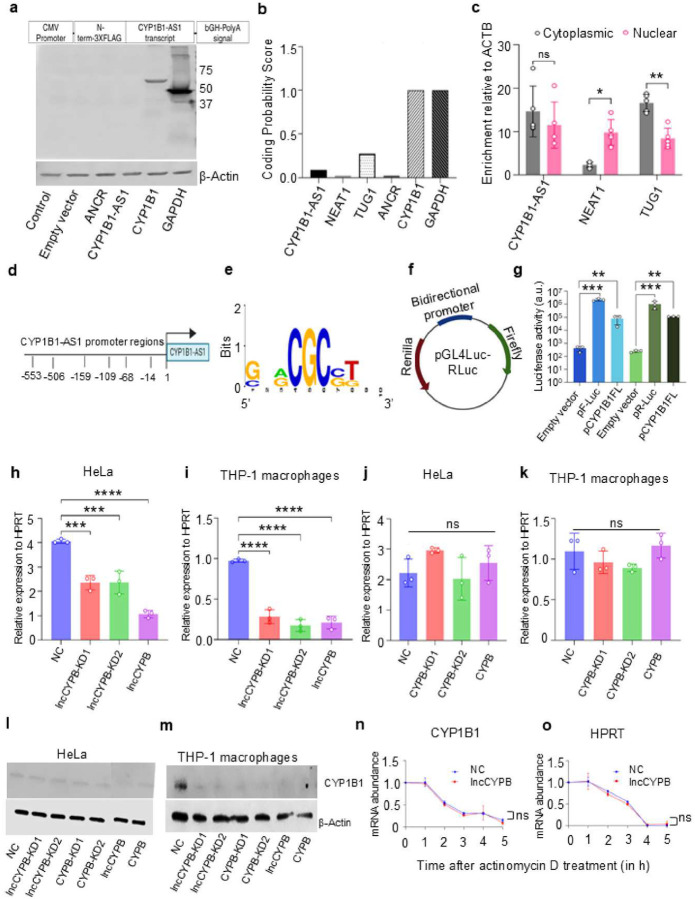
CYP1B1-AS1 is transcribed from a shared bidirectional promoter and regulates CYP1B1 in *cis*. (a) Coding potential was assessed by transfecting pCDNA-CYP1B1-AS1 into HEK293T cells and immunoblotting for FLAG antibody (b) The Coding Potential Assessment Tool (CPAT) predicts a very low coding potential for CYP1B1-AS1. TUG1, NEAT1, ANCR, and MALAT1 serve as control lncRNAs, while GAPDH is the positive control for mRNA. (c) qPCR analysis of RNA from nuclear and cytoplasmic fractions to analyze the localization of CYP1B1-AS1 in THP-1 cells. Expression was normalized to ACTB and compared to the cytoplasmic fraction. NEAT1 and TUG1 are used as nuclear- and cytoplasmic-enriched controls, respectively. (d) A schematic of the transcription factor binding sites (TFBS) of AHR is predicted upstream of CYP1B1-AS1 transcription start site (TSS; +1) by JASPAR and Expasy Eukaryotic promoter database (EPD) (P-value < 0.001). (e) Motif binding sequence logo of AHR. (f) Schematic of the reporter assay to study bidirectional promoter activity. (g) Luciferase reporter assay demonstrating bidirectional promoter activity of CYP1B1-AS1 (P_*CYP1B1-AS1*_). F-Luc/FL represents firefly luciferase activity and R-Luc/RL represents renilla luciferase activity. pF-Luc and pR-Luc are positive controls for P_*ACTB*_. (h-i) qPCR analysis showing CYP1B1 expression in CYP1B1-AS1 knockdown in: (h) HeLa and (i) THP-1 macrophages. (j-k) qPCR analysis showing CYP1B1-AS1 expression in CYP1B1 knockdown in: (j) HeLa cells, and (k) THP-1 macrophages. Gene expression was normalized to HPRT and compared to cells treated with negative control siRNA (NC). KD1 and KD2 represent different siRNA mediated knockdowns and their combination used for target silencing, designated as lncCYPB or CYPB. (l-m) Immunoblot analysis of CYP1B1 levels in lncCYPB cells: (l) HeLa cells, and (m) THP-1 macrophages. β-Actin serves as the loading control. (n-o) mRNA decay assay of: (n) CYP1B1, and (o) HPRT in NC and lncCYPB cells after actinomycin D treatment upto 5 h. The half-lives (t_1/2_) of mRNA were calculated using one-phase decay analysis from 2^(-ΔCT)^ values of transcripts. For all data, n=3 and, error bars indicate mean ± SD. Statistical significance: *, P < 0.05; **, P < 0.01; ***, P < 0.0001; ****, P < 0.0001; ns, not significant, P ≥ 0.05; (g) two-way ANOVA; (h-k) one-way ANOVA.

CYP1B1-AS1 is located on the sense strand of human chromosome 2, spans 1.7Kb, and is a polyadenylated lncRNA (Fig. S7a). Open reading frame (ORF) analysis identified short ORFs within the CYP1B1-AS1 sequence, but BLAST analysis revealed no similarity to known protein sequences. To further assess its coding potential, CYP1B1-AS1 was cloned into a CMV promoter-driven 3X-FLAG tagged pCDNA vector (pCDNA-FLAG, Table S2), transfected into HEK293T cells, and analyzed via immunoblotting for FLAG expression. No detectable protein signal was observed, confirming its non-coding nature (Fig. 3a). This result was further supported by the Coding Potential Assessment Tool (CPAT)^31^ (Fig. 3b). LncRNA function is often linked to its subcellular localization^23, 32^. Using cellular fractionation assays, we found that CYP1B1-AS1 was equally distributed between the nuclear and cytoplasmic fractions (≈1.2-fold enrichment in the nucleus compared to the cytoplasm, P ≥ 0.05) (Fig. 3c).

Previous studies have shown that lncRNAs, particularly those transcribed from bidirectional promoters, often regulate nearby protein-coding genes in *cis*^23, 33^. Our results revealed that CYP1B1-AS1 and CYP1B1 exhibited similar expression patterns, positively correlating with the AHR TF during Cb infection (Fig. 1 and 2). This prompted further investigation into shared regulatory elements. We analyzed the CYP1B1-AS1 promoter region for AHR transcription factor binding sites (TFBS) using UCSC (https://genome.ucsc.edu/cgi-bin/) and Expasy eukaryotic promoter database (https://epd.expasy.org/epd/) prediction tools. The region upstream of TSS contained AHR motifs [Fig. 3d, Fig. 3e, and Fig. S7 (b-c)].

To confirm the bidirectional nature of the promoter, a dual-luciferase reporter assay was performed by cloning the promoter into pGL4-Luc-Rluc (Fig. 3f). The assay showed that the promoter activated both Renilla (direction of CYP1B1) and firefly (direction of CYP1B1-AS1) luciferase by at least 2.5-fold compared to the empty vector, indicating it is a functional bidirectional promoter (Fig. 3g). Furthermore, the region exhibited histone modification markers (H3K4me1, H3K4me3, and H3K27Ac), indicative of active transcription. UCSC genome browser predictions also showed Pol II and DNase I hypersensitivity signals, suggesting chromatin modifications in the promoter region (Fig. S7d)^23^. CpG islands are often found in bidirectional promoters^23, 34^. Our EMBOSS CpGplot (https://www.ebi.ac.uk/jdispatcher/seqstats/emboss_cpgplot) analysis also showed a higher observed-to-expected CpG ratio, and a GC content above 50% (Fig. S7e). These findings demonstrate that CYP1B1-AS1 and CYP1B1 co-expression is driven by a shared bidirectional promoter.

Next, we investigated whether CYP1B1-AS1 regulates CYP1B1 expression in *cis*. We performed knockdowns using combinations of siRNA (listed in methods) targeting CYP1B1-AS1 (lncCYPB-KD1, lncCYPB-KD2, lncCYPB) and CYP1B1 (CYPB-KD1, CYPB-KD2, CYPB), with GAPDH siRNA as a positive control (PC) and a negative control siRNA (NC) for comparison. Knockdown efficiency was highest in lncCYPB (≈90% in HeLa cells and 67% in THP-1 macrophages) and CYPB (≈90% in HeLa cells and 70% in THP-1 macrophages) -silenced cells [Fig. S8(a-h)]. Knocking down CYP1B1-AS1 significantly reduced CYP1B1 mRNA expression by ≈4.57-fold in HeLa (Fig. 3h) and ≈4.95-fold in THP-1 macrophages (Fig. 3i). In contrast, CYP1B1 knockdown did not affect CYP1B1-AS1 expression in either HeLa (Fig. 3j) or THP-1 macrophages (Fig. 3k), indicating that CYP1B1-AS1 regulates CYP1B1 in *cis* but not *vice versa*. Immunoblot analysis of CYP1B1-AS1 knockdown cells showed an ≈80% reduction in CYP1B1 protein levels, confirming that this regulatory effect occurs at the protein level [Fig. 3 (l–m)].

To determine post-transcriptional regulation of CYP1B1 by CYP1B1-AS1, we performed a mRNA decay assay. The cells were treated with actinomycin D to inhibit global transcription in lncCYPB and NC cells. The knockdown of CYP1B1-AS1 did not affect the half-life of CYP1B1 mRNA up to 5 h of treatment (Fig. 3n), nor did it impact HPRT mRNA (control) (Fig. 3o). In conclusion, these data demonstrated that CYP1B1-AS1 doesn’t regulate the mRNA stability of CYP1B1 and regulates its expression from a bidirectional promoter in *cis*.

### CYP1B1-AS1 promotes Cb replication in macrophages via modulation of host cell response

**Fig 4.**
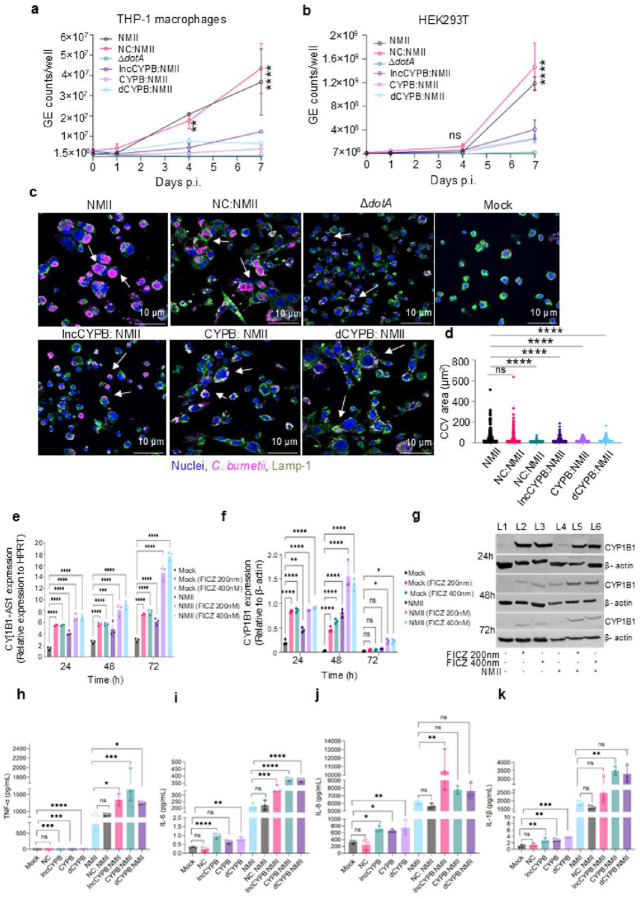
CYP1B1-AS1 and CYP1B1 are transcriptionally activated by AHR, and their knockdown reduces cell permissiveness to Cb replication. (a-b) Bacterial growth was assessed by quantifying genomic equivalents (GE) over a 7-day time course. Cells without knockdown, infected with Cb and CbA, are designated as NMII and Δ*dotA*, respectively. Knockdown of CYP1B1 (CYPB), CYP1B1-AS1 (lncCYPB), both genes (dCYPB), and NC cells infected with Cb are designated as CYPB: NMII, lncCYPB: NMII, dCYPB: NMII, and NC: NMII, respectively. GE counts in (a) THP-1 macrophages at MOI of 50, and (b) in HEK293T cells at an MOI of 100. Results are expressed as means from three technical replicates of three independent experiments. (c) Immunofluorescence staining and representative images of Cb-infected THP-1 macrophages at 5 days p.i. Nuclei (blue), LAMP1 (green), Cb and CbA (magenta); scale bar: 10 μM. (d) Quantitative measurements of CCV (*Coxiella* containing vacuoles) using ImageJ, QuPath, and Labkit algorithms, with each LAMP1^+^ bacterial compartment representing an individual CCV. Data are shown as mean ± SD of at least 100 CCVs per cell type. (e) qPCR analysis of CYP1B1-AS1 expression in cells treated with FICZ at concentrations of 200 nM and 400 nM, and infected with Cb. Expression was normalized to HPRT and compared to Mock and Mock treated with FICZ as controls. (f) Densitometry plot analysis of CYP1B1 levels using ImageJ. (g) Representative immunoblot of CYP1B1 expression in Mock and NMII cells treated with FICZ, with β-Actin as the loading control. ELISA quantification of: (h) TNF-α, (i) IL-6, (j) IL-8, and (k) IL-1β. For all data, n=3, and error bars indicate mean ± SD. Statistical significance: *, P < 0.05; **, P < 0.01; ***, P < 0.0001; ****, P < 0.0001; ns, not significant, P ≥ 0.05. Statistical tests used: (d, h-k) one-way ANOVA; (e, g) two-way ANOVA.

CYP1B1-AS1-dependent upregulation of CYP1B1 prompted us to investigate the role of these genes in Cb pathogenesis. Growth curve analyses revealed a significant reduction in Cb load (approximately 2-fold decrease; Fig. 4a) in knockdown-infected cells; lncCYPB:NMII, CYPB:NMII, and dCYPB:NMII (dual knockdown of CYP1B1-AS1 and CYP1B1; Table S3) cells compared to NC and non-siRNA treated cells infected with Cb (designated as NC: NMII, NMII). Similar results were observed in HEK293T knockdown cells with >85% knockdown efficiency, showing a 2-fold reduction in Cb growth by 7-day p.i. (Fig. 4b). Furthermore, CCVs, stained for LAMP-1 and Cb antibodies, demonstrated that CYP1B1-AS1 and CYP1B1 are necessary for CCV expansion (Fig. 4c). The average size of the CCVs were significantly smaller in knockdown cells which corroborated with the growth curve data (Fig. 4d).

Next, we ectopically overexpressed CYP1B1-AS1 and CYP1B1 in the pcDNA3.1/Zeo (+) vector (designated as pCYP1B1-AS1 and pCYP1B1; Table S2) to assess their impact on Cb replication. No detectable change in bacterial load was observed in HeLa overexpressing these genes (Fig. S9a). However, pCYP1B1 expression in HEK293T cells showed increased Cb replication at 7-day p.i. (Fig. S9b), potentially due to differences in cell type or transfection efficiency. Notably, CYP1B1-AS1 overexpression did not alter Cb survival or CYP1B1 transcript levels in any cell type tested [Fig. S9 (c-d)], indicating that CYP1B1-AS1 does not regulate CYP1B1 in *trans*.

AHR is known to play a critical role in regulating intestinal homeostasis, often activated by tryptophan metabolites^29^. Additionally, AHR can be modulated independently of ligands via nucleocytoplasmic shuttling during oxidative stress, leading to the activation of target genes, including members of the cytochrome P450 family such as CYP1A1 and CYP1B1^30, 35^. While the role of AHR has been explored in several viral, bacterial, and parasitic infections, its regulation in Cb pathogenesis remains unstudied.

In this study, we identified AHR as a TF for CYP1B1-AS1 and CYP1B1 activation. We examined its role by treating infected cells with the AHR agonist 6-Formylindolo [3,2-b] carbazole (FICZ), a tryptophan derivative, at concentrations of 200 nM and 400 nM. FICZ-treated mock cells exhibited a 4-fold increase in CYP1B1-AS1 expression, while FICZ-treated infected cells showed an ≈8-fold increase in CYP1B1-AS1 compared to untreated controls (Fig. 4e) from 24 h to 72 h p.i., as confirmed by qPCR analysis. Concurrently, immunoblots and densitometry analysis showed an ≈5-fold increase in CYP1B1 protein levels in FICZ-treated infected cells compared to controls (Fig. 4f, 4g). AHR transcript levels also showed a Cb- and FICZ-dependent increase of ≈2 to 3-fold compared to the control (Fig. S9e). However, no significant changes in Cb intracellular replication were observed in FICZ-treated THP-1 cells (Fig. S9f), suggesting that AHR’s role in the infection may involve modulation of multiple signaling pathways. These results indicate that AHR is activated in response to Cb infection and transcriptionally regulates CYP1B1-AS1 and CYP1B1, potentially contributing to immune modulation and promoting Cb replication within the host.

### CYP1B1-AS1 and CYP1B1 regulates inflammation during Cb infection

Cb activates or dampens multiple signaling pathways in host immune cells, modulating inflammatory cytokine production during infection^36, 37, 38^ The T4SS activity of Cb is primarily known to suppress the inflammatory cytokines^39^, while lncRNAs are increasingly recognized as key regulators of inflammation and immune responses^19, 40^.

Building on these findings, we investigated the role of CYP1B1-AS1 and CYP1B1 in modulating the inflammatory response during Cb infection. We observed significantly elevated TNF-α levels in lncCYPB, CYPB, and dCYPB knockdown cells, both under control conditions and during infection (Fig. 4h). Additionally, lncCYPB cells exhibited significantly increased levels of IL-6, IL-8, and IL-1β [Fig. 4(i–k)], indicating that CYP1B1-AS1 plays a critical role in regulating inflammation during Cb infection. The expression of other cytokines was also quantified in the knockdown cells and is shown in Fig. S10(a-f). ELISA results of the cytokines corroborated well with transcript expression of TNF-α, IL-6, IL-8 and IL-1β [Fig. S10 (g-l)].

Notably, with the exception of TNF-α, CYP1B1 knockdown resulted in elevated levels of IL-6, IL-8, and IL-1β, independent of CYP1B1-AS1 regulation, as observed in CYPB and dCYPB cells [Fig. 4(i–k)]. This indicates that while CYP1B1-AS1 is essential for dampening inflammatory cytokines during Cb infection, via or independent of CYP1B1.

Collectively, these findings demonstrate that CYP1B1-AS1 and CYP1B1 may have overlapping roles in controlling inflammation during infection, but they likely employ distinct mechanisms to modulate the host immune response.

### CYP1B1-AS1 and CYP1B1 knockdown generates mitochondrial ROS and induces membrane depolarization

A decrease in CYP1B1 activity has been linked to increased ROS-induced oxidative stress, inflammatory disorders, and disruption of mitochondrial homeostasis^21^. We do not fully understand why silencing CYP1B1-AS1 or CYP1B1 differentially disrupts the cytokine milieu, leading to inflammation and reduced intracellular Cb survival.

**Fig 5.**
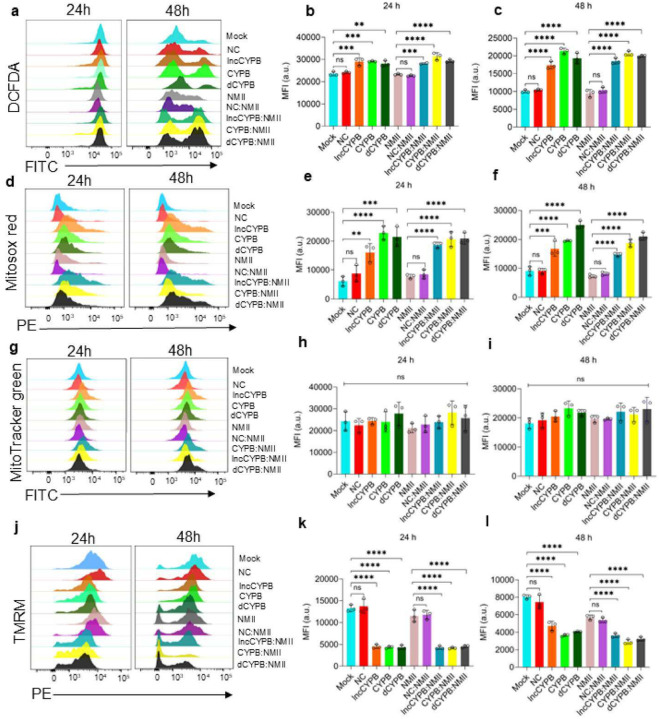
CYP1B1-AS1 and CYP1B1 knockdown generates mitochondrial reactive oxygen species (ROS) and promotes mitochondrial dysfunction. (a) Representative flow cytometry analysis of control and infected cells stained with DCFDA. (b-c) Mean fluorescence intensity of intracellular ROS produced in DCFDA-stained cells at: (b) 24 h p.i. and (c) 48 h p.i. (d) Mitochondrial ROS production was assessed by flow cytometry staining using MitoSOX^™^ red staining. (e-f) Mean fluorescence intensity of mitochondrial ROS in MitoSOX^™^ red-stained cells at: (e) 24 h p.i. and (f) 48 h p.i. (g) Mitochondrial mass of cells were assessed by flow cytometry analysis of MitoTracker^™^ Green-stained cells. (k-l) Mean fluorescence intensity of MitoTracker^™^ Green-stained cells at: (h) 24 h p.i. and (i) 48 h p.i. (j) Mitochondrial membrane potential was measured by flow cytometry analysis of MitoProbe^™^ TMRM-stained cells. (k-l) Mean fluorescence intensity of mitochondrial membrane potential of MitoProbe^™^ TMRM-stained cells at: (k) 24 h p.i. and (l) 48 h p.i. For all data, n=3, and error bars indicate mean ± SD. Statistical significance: **, P < 0.01; ***, P < 0.0001; ****, P < 0.0001; ns, not significant, P ≥ 0.05; one-way ANOVA.

Therefore, to explore these findings further, we investigated the impact on mitochondrial function and its connection to infection outcomes. To define the repertoire of free radicals in CYP1B1-AS1 and CYP1B1 knockdowns during Cb infection, we used DCFDA and the mitochondrial-specific superoxide indicator mitoSOX for flow cytometry analysis. Results showed significantly increased DCFDA fluorescence in lncCYPB, CYPB, and dCYPB cells at 24 h and 48 h p.i., both under control conditions and during infection [Fig. 5(a–c)]. Mitochondrial ROS was also measured using mitoSOX and mitotracker green to stain active mitochondria. The analysis revealed significantly elevated mitochondrial ROS, as evidenced by increased mitoSOX fluorescence [Fig. 5(d–f)], while mitochondrial mass remained consistent, indicating that ROS was generated from active mitochondria [Fig. 5(g–i)].

Since elevated mitochondrial ROS can disrupt mitochondrial function leading to altered membrane potential^41^, we measured mitochondrial membrane potential using tetramethylrhodamine methyl ester (TMRM). TMRM is a lipophilic cell-permeant, cationic dye, that is readily sequestered by healthy mitochondria. TMRM fluorescence intensity was decreased by ≈2-fold at 24 h and 1.5-fold at 48 h p.i. in lncCYPB, CYPB, and dCYPB cells compared to controls [Fig. 5(j–l)], indicating mitochondrial membrane depolarization.

These findings demonstrate that CYP1B1-AS1 and CYP1B1 are critical for maintaining mitochondrial function and regulating ROS during Cb infection.

### CYP1B1-AS1 and CYP1B1 regulates mitochondrial ROS and inhibits apoptosis

**Fig 6.**
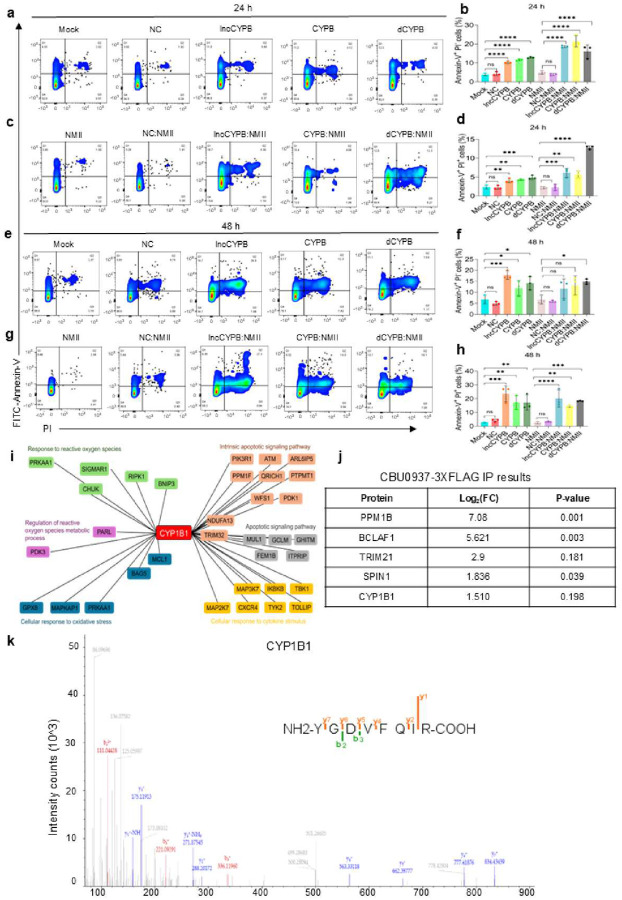
CYP1B1-AS1 and CYP1B1 regulate mitochondrial ROS and inhibits apoptosis. Fluorescence was detected using flow cytometry to analyze the populations of early apoptotic (Annexin-V^+^ PI^−^; Q1), late apoptotic (Annexin-V^+^ PI^+^; Q2), dead (PI^+^; Q3), and non-apoptotic (Annexin-V^−^ PI^−^; Q4) cells. (a-d) Flow cytometry analysis of cells stained with Propidium Iodide (PI) and FITC-Annexin-V at 24 h p.i. (a) Representative flow cytometry of control cells at 24 h p.i. (b) Mean fluorescence intensity of the early apoptotic population at 24 h p.i. (c) Representative flow cytometry of Cb-infected cells at 24 h p.i. (d) Mean fluorescence intensity of late apoptotic population at 24 h p.i. (e-h) Flow cytometry analysis of cells stained with PI and FITC-Annexin-V at 48 h p.i. (e) Representative flow cytometry of control cells at 48 h p.i. (f) Mean fluorescence intensity of the early apoptotic population (g) Representative flow cytometry of Cb-infected cells at 48 h p.i. (h) Mean fluorescence intensity of late apoptotic population at 48 h p.i. (i) Protein-protein interaction (PPI) analysis map of IP-enriched proteins of 3×FLAG-CYP1B1, manually constructed and assigned to functional groups based on biological pathways. (k) Proteins identified by quantitative MS/MS analysis following IP of CBU_0937 tagged with C-terminal 3XFLAG (CBU0937–3XFLAG). Gene names of selected proteins and corresponding Log_2_(fold-change; FC) and P-value of each protein are listed. (I) Annotated LC-MS/MS spectrum of m/z 499.258 from CBU0937–3XFLAG IP confirming the presence of CYP1B1. Missing annotation on the peptide sequence denotes lack of detection of that particular b/y ion fragment. For panels (a-h), n=3, and error bars indicate mean ± SD. Statistical significance: *, P < 0.05; **, P < 0.01; ***, P < 0.0001; ****, P < 0.0001; ns, not significant, P ≥ 0.05; (b, d, f and h) one-way ANOVA.

We hypothesized that mitochondrial dysfunction on CYP1B1-AS1 and CYP1B1 knockdown triggers apoptosis. To explore this, we measured Annexin-V and Propidium Iodide (PI) accumulation. lncCYPB, CYPB, and dCYPB cells displayed increased Annexin-V accumulation, while lncCYPB:NMII, CYPB:NMII, and dCYPB:NMII cells showed significantly higher Annexin-V^+^ and Annexin-V^+^PI^+^ populations at 24 h p.i. compared to mock and infected controls [Fig. 6(a–d)]. By 48 h p.i., these knockdown cells progressed further toward late apoptosis, as evidenced by an increased Annexin-V^+^PI^+^ population [Fig. 6(e–h)], with infected cells showing increased cell death, as observed from increased Annexin-V^−^ PI^+^ signal compared to controls [Fig. S11(a-b)].

Having shown that CYP1B1-AS1 enhances CYP1B1 expression to regulate mitochondrial ROS and apoptosis in Cb pathogenesis, we next investigated the mechanisms linking CYP1B1 upregulation to these effects. We overexpressed 3XFLAG-CYP1B1construct (pcDNA-CYP1B1, Table S2) in HEK293T cells and infected them with Cb, using 3XFLAG-GFP (pcDNA-eGFP) as a control. A schematic of the workflow is provided in Fig. S11c. This design was chosen for two reasons: (a) CYP1B1-AS1 overexpression did not drive CYP1B1 expression in *trans* (b) to examine proteins that are specifically enriched by CYP1B1 upregulation during Cb infection.

Based on this hypothesis, we performed immunoprecipitation (IP) experiments and identified associated proteins via mass spectrometry. The identified peptides, associated data, and enriched pathways are presented in Data S6 and S7. After filtering out common background proteins from the 3XFLAG-GFP datasets, we compared the enriched proteins in CYP1B1-overexpressing and CYP1B1-overexpressing-infected samples to identify commonly associated pathways (Data S6). Functional enrichment analysis revealed that these proteins were primarily associated with the ROS response and negative regulation of apoptosis pathways [Fig. S12 (a-d), Data S7]. Notably, CYP1B1 overexpression was linked to pathways including TNF signaling, p38/MAPK, mTOR, AMPK, and PI3K/AKT. This enrichment of pathways alongside CYP1B1 is particularly intriguing, as CYP1B1 is a downstream product of AHR activation, which supports the phenotypes observed in our study. To further illustrate, a partial map was constructed to demonstrate the association of CYP1B1 with proteins involved in ROS and apoptosis regulation during pathogenesis (Fig. 6i; Data S8).

Importantly, our mass spectrometry analysis identified CBU_0937 as a potential interactor with 3XFLAG-CYP1B1, albeit at a medium confidence level. However, due to the high abundance ratio of eukaryotic to bacterial proteins limited our ability to obtain a reliable m/z spectrum for the CBU_0937 peptide. To investigate this finding, we constructed a plasmid expressing a C-terminal-3XFLAG tagged CBU_0937 (pCDNA-CBU0937, Table S2) and conducted an IP from HEK293T transfected cells, using a GFP control (pCDNA-GFPc, Table S2) for comparison. CYP1B1 emerged as one of the potential interactors of CBU_0937 among other candidates (Fig. 6j, Data S9). The representative m/z spectra and associated peptide for CYP1B1 are shown in Fig. 6k.

**Fig 7.**
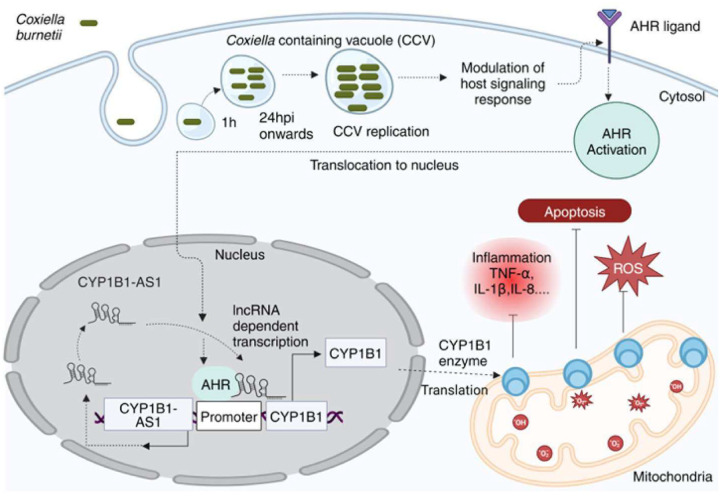
Proposed mechanism of CYP1B1-AS1 dependent regulation of CYP1B1 during Cb infection. During Cb infection transcription factor AHR is activated. AHR transcriptionally activates both CYP1B1-AS1 and CYP1B1 from a shared bidirectional promoter. This activation leads to the regulation of CYP1B1 transcripts, which is dependent on CYP1B1-AS1 expression through a *cis* mechanism within the local genome. The subsequent transcriptional regulation of CYP1B1 results in increased turnover of the CYP1B1 enzyme. CYP1B1, a cytochrome P450 1B1 enzyme that is enriched in the mitochondria of the cell. It plays a critical role in maintaining mitochondrial redox homeostasis by regulating ROS, thereby reducing ROS-mediated inflammation and inhibiting apoptosis. This regulatory mechanism may promote increased cell survival during Cb infection.

Collectively, our results demonstrate that Cb infection activates AHR, which induces CYP1B1-AS1 expression, subsequently enhancing CYP1B1 transcription in *cis*. Additionally, we identify *Coxiella* protein CBU_0937 as an interactor with CYP1B1, potentially influencing Cb pathogenesis and survival. Cell death modality usage is a crucial strategy for pathogenic infections, and Cb exploits this unique host lncRNA-mediated reprogramming of the signaling landscape to modulate infection outcomes (Fig. 7).

## Discussion

The role of CYP1B1-AS1 and CYP1B1 has been studied in various cancers^42, 43, 44, 45^, but this study is the first to explore their regulation of host immune responses during Cb infection. Through RNA-seq analysis across sixteen different host-microbe interactions, we identified CYP1B1-AS1 as a lncRNA biomarker for Cb infection. Among lnc-DKK2 and CYP1B1-AS1, CYP1B1-AS1 stood out for its high correlation with the Cb-infected host transcriptome, making it a promising candidate for further investigation into host-pathogen interactions.

This study also sheds light on other infection-specific lncRNAs across various pathogens demonstrating the broad relevance of lncRNA in modulating host immune responses. For example, the dysregulation of NDUFV2-AS1, which is located antisense to NDUFV2, a gene previously implicated in increased ROS production and apoptosis^46, 47^—underscores the critical role of NDUFV2-AS1 in regulating mitochondrial function and inflammation in EHECΔ*stx* infection. Further investigation into the functions of these lncRNAs in infection and immune modulation would be of significant interest.

LncRNAs are known to function through multiple mechanisms^15^. The transcriptional regulation of CYP1B1 by CYP1B1-AS1 from a bidirectional promoter in *cis* is a promising step toward understanding how CYP1B1-AS1 modulates immune responses to promote Cb pathogenesis. As demonstrated through CpG and dual reporter luciferase analysis, the bidirectional promoter region of these genes also lacks a standard TATA box, a characteristic feature of many bidirectional promoters in the eukaryotic genome^23, 34^. LncRNAs transcribed from such promoters often show co-expression with their neighboring protein-coding genes^23, 34^, which was observed for CYP1B1-AS1 and CYP1B1 at various time points during infection. However, this does not rule out the possibility that CYP1B1-AS1 also regulates genes at more distal sites, which warrants further exploration. Although CYP1B1-AS1 and CYP1B1 primarily drive similar functions, our mass spectrometry data suggest that they may also engage in distinct regulatory pathways. This is particularly significant because many genes affected by Cb infection at both translational and transcriptional levels are involved in survival pathways, dysregulated autophagy, and apoptosis mechanisms.

One key finding of our study is the activation of the AHR pathway leading to upregulation of CYP1B1-AS1 and CYP1B1 in Cb-infected macrophages. The activation of AHR is known to reduce inflammatory cytokines TNF-α, IL-6, IL-12 and IL-17 responses in macrophages^29, 30, 48, 49^. Cb’s T4SS also been reported to dampen IL-17 signaling to protect against ROS-induced bactericidal activity in macrophages (36). This study highlights the role of the AHR signaling pathway in regulating Cb pathogenesis and opens avenues for further investigation into its influence in *Coxiella* biology, particularly in the regulation of IL-17-mediated ROS responses in macrophages^36^.

Knockdown of CYP1B1-AS1 and CYP1B1 resulted in an elevated inflammatory response, particularly for TNF-α, IL-6, and IL-1β, which Cb is previously known to dampen in order to promote its intracellular survival^39, 50, 51, 52^. Elevated TNF-α has been previously shown to limit Cb infection locally and systemically^53, 54^. These findings correlate with the reduced intracellular Cb load in CYP1B1-AS1 and CYP1B1-silenced cells. Furthermore, TNF-α, IL-6, and IL-1β are known to synergize in triggering apoptosis during inflammation and infection^55, 56^. Collectively, our results reaffirm the crucial role of CYP1B1-AS1 in regulating inflammatory responses and mitochondrial functions during Cb infection.

Our data show that CYP1B1-AS1 regulates mitochondrial ROS production via CYP1B1, with knockdowns of either gene leading to disrupted mitochondrial function and increased cell death. The phenotypes associated with CYP1B1-AS1 were largely attributed to the downregulation of CYP1B1, which makes CYP1B1 a crucial checkpoint in antimicrobial defense and the regulation of programmed cell death. This connection between CYP1B1-AS1, CYP1B1, and apoptosis is supported by our mass spectrometry data indicating regulation of intrinsic apoptosis pathways. While we have established a connection between CYP1B1-AS1/CYP1B1 in regulating mitochondrial ROS production and mitigating cell death, the exact mechanisms and specific cell death modalities involved remain unclear. Further studies are needed to identify the molecular factors driving CYP1B1-AS1/CYP1B1-mediated regulation of programmed cell death.

A promising candidate for such a role is CBU_0937, a mitochondrial-targeted *Coxiella* protein identified as a potential CYP1B1 interactor. CBU_0937 possesses a sec-dependent signal peptide, a β-sheet topology, and a LbtU porin domain, displaying high sequence homology to known bacterial porins^57, 58^. Bacterial β-barrel porins, such as PorB and Omp85 from *Neisseria* species, have been previously shown to interact with mitochondrial membranes, either promoting cell survival or sensitizing cells to apoptosis^59, 60^. Although CBU_0937’s role in Cb intracellular pathogenesis is debated^58, 61^, our findings and existing literature suggest that CBU_0937 could interact with host mitochondrial proteins such as CYP1B1 during infection. Further studies are warranted to clarify the specific nature of this interaction and its impact on host signaling responses.

In summary, our study contributes to the emerging model of the regulatory roles of lncRNAs and highlight the adaptive strategies employed by obligate intracellular pathogens to manipulate host defenses, including the flexible utilization of mitochondrial enzymes. Overall, our observations highlight CYP1B1-AS1 as a central player in modulating inflammation, ROS production, and cell death by regulating CYP1B1. This discovery offers valuable insights into a novel immune signaling pathway, with potential implications for the development of RNA-based therapeutics targeting Cb infections.

## Materials and Methods

### *C. burnetii* strains and culture conditions

All Cb strains used in this study were derived from Nine Mile phase II (NMII), strain RSA439 (Cb), clone 4 (Table S1). Strains were cultured for 7 days in ACCM-2 (Sunrise Science Products) at pH 4.75, 37°C in 2.5% O_2_ and 5% CO_2_ in a tri-gas incubator (New Brunswick^™^, Eppendorf). Bacterial cultures were centrifuged at 4000 rpm for 20 mins and the pellet was re-suspended in Roswell Park Memorial Institute (RPMI) 1640 medium (Cytiva HyClone^™^), then stored as described previously^62^. For Cb Nine Mile phase I (NMI) clone 7 (RSA493) culture, the strain was grown in embryonated yolk sacs, and purified using density-gradient centrifugation as described previously^12^. Genome equivalents (GE) for each bacterial stock were calculated using qPCR as described below. Experiments with *C. burnetii* NMI were performed in biosafety level 3 (BSL3) facility at Texas A&M University Health Science Center, USA.

### Bacterial strains and culture conditions

The bacterial strains used in this study include Ec5, EcT, EcN, Bs, Fn, Pa, Sa, STm, Rr, Ml, Li, Ef and Bmv (Table S1). Sa, Bmv, Pa and Fn were grown for 20 h (overnight) in Tryptic soy broth (TSB; Millipore-Sigma) medium at 37°C and 220 rpm in a shaker-incubator (MaxQ^™^ Thermo Scientific^™^). Ef and Li were cultured overnight in Brain heart infusion (BHI; Millipore-Sigma) broth at 37°C and 220 rpm. EcT, EcN and STm were cultured in Luria-Bertini (LB; MP Biomedicals) medium at 37°C and 220 rpm. Ec5, Bs, Li, Rr and Ml were also cultured in LB medium at 37°C and 220 rpm. For spatio-temporal gene expression study *in vitro*, bacterial strains Bmv, Li and STm were cultured in their respective media as described above. Lp was cultured in buffered charcoal yeast extract (BCYE; Millipore-Sigma) broth with legionella growth supplements at 37°C and 220 rpm overnight. The overnight cultures were sub-cultured until early logarithmic phase OD_600_ to 0.6, centrifuged at 4000 rpm for 10 minutes, and the pellets were washed with PBS (Cytiva HyClone^™^) three times before resuspension in PBS for mammalian cell infection.

### Mammalian cell culture

THP-1 monocytes were cultured in RPMI 1640 with 10% fetal bovine serum (FBS; Avantor^®^) to confluence in 5% CO_2_ incubator (Nuaire). The cells were then seeded in appropriate cell culture dish and treated with 100 ng/mL PMA (InvivoGen) to differentiate into macrophages^63^. After 24 h of PMA treatment, the medium was removed, and cells were allowed a 24-hour resting phase before experiments. HEK293T and HeLa cells were cultured in Dulbecco’s Modified Eagle’s Medium (DMEM; Sigma-Aldrich) with 10% FBS to confluence in a 5% CO2 incubator for subsequent experiments.

### Mammalian cell infections for RNA-seq and transcript analysis

For RNA-seq, inoculum was prepared as described previously or from frozen stocks (for Cb infections) to infect THP-1 macrophages at an MOI of 50 for 1 h. Briefly, THP-1 cells were differentiated into macrophages in 6-well plates (Greiner) at a density of 1 ×10^6^. The inoculum containing RPMI media was added to the cells, which were centrifuged at 500 × g for 10 mins to synchronize bacterial uptake. Cells were collected 1 h p.i., snap-frozen in liquid nitrogen, and processed for RNA isolation and sequencing as described below.

For spatio-temporal gene expression studies, THP-1 macrophages and HeLa cells were infected with Cb and other strains at an MOI of 50 or 100. Briefly, Cb infections were carried out from frozen stocks into cells seeded at density of 1 × 10^6.^ After 1 h, the medium was replaced with fresh medium without antibiotics. Cells were collected at specified time points for RNA isolation and qPCR analysis. For Lp, Bmv, Li and STm infections, THP-1 macrophages were maintained in RPMI with 10% FBS, without antibiotics. Infections were performed at an MOI of 50, followed by centrifugation at 500 × g for 10 mins and incubation for 1 h. Cells were then washed, and medium containing 100 μg/mL gentamicin (Sigma-Aldrich) was added for 1 h to kill extracellular bacteria. Following gentamicin treatment, cells were maintained in antibiotic-free medium and collected at specified time points for RNA isolation and qPCR.

### RNA sequencing and data analysis

The RNA-seq experiment included 64 samples: 4 biological replicates per condition, comprising 64 infected and 4 uninfected controls (Mock). THP-1 macrophages were infected with 16 different strains as described above. Total RNA was isolated using Trizol^™^ Reagent (Invitrogen^™^) and Direct-zol RNA Miniprep Plus kits (Zymo Research), followed by 1 h DNase treatment. RNA quality and quantity were assessed using the 4200 TapeStation system (Agilent Technologies).

RNA-seq and library preparation were performed by Texas A&M AgriLife Genomics and Bioinformatics Service, and sequencing was conducted on the Illumina HiSeq4000 platform using 2 × 150-bp paired-end reads. Quality control of the raw data was carried out using the NGS QC Toolkit, ensuring that at least 70% of the bases had a Phred quality score of ≥ 30. Clean FASTQ data were mapped to the *Homo sapiens* NCBI reference genome (GCF_000001405.40) with annotation from the GENCODE database (Release 30, GRCh38.p12) using STAR (v 2.5.4b). This generated 35.9–83.4 million aligned reads per sample, representing 73–87% of the total sequence reads per sample. Uniquely mapped reads were used to count the number of reads per gene, ensuring that each read was mapped to only one gene based on its genomic location. Both ends of the paired-end reads were checked for overlaps. Alignment properties and raw counts from the RNA-seq analysis are provided in Dataset S3.

### Transcriptome data processing and differential expression analysis

RNA-seq data were normalized using the DESeq function in the DESeq2 R package (v 1.36.0), adjusting for known biological conditions of the pathogen samples^64^. Variance stabilizing transformation (VST)^65^ was applied to the data, followed by differential expression analysis using DESeq2 R package. Relative transcript expression was calculated as Reads Per Kilobase per Million mapped reads (RPKM), with fold change log_2_-transformed. Statistical significance was determined using Benjamini and Hochberg’s correction to adjust P-values. Genes with a log_2_ fold change (log_2_FC) >1 or <−1 and adjusted P < 0.05 were considered DE. Principal component analysis (PCA) was performed using the R prcomp base function, with the top 1000 most variable genes as input. Visualizations, including box plots, volcano plots, and hierarchical clustering, were generated using R 3.6.0 (www.r-project.org).

### Prediction and annotation of lncRNA targets

The human lncRNA catalog used in this study was extracted from the GENCODE human gene annotation database. Identified lncRNAs were reclassified as lincRNA, anti-sense lncRNA, or sense lncRNA based on their biotypes in the GENCODE catalog. A list of anti-sense or sense-overlapping lncRNAs was filtered using manually annotated gene annotations from GENCODE. Adjacent protein-coding genes within 5 kb of lncRNAs were predicted as their target genes based on their genomic proximity to lncRNAs.

### Identification of unique DE-lncRNA in Cb infection

A list of DE-lncRNAs for each strain was generated and compared to those identified in Cb infection. An UpSet plot from the concatenated list of DE-lncRNAs in all strains was generated using the R package “UpSetR” (v 1.4.0) which displayed unique DE-lncRNAs for Cb and other strains.

### Functional enrichment and pathway analysis of lncRNAs

Functional enrichment analysis of DE-lncRNA and mRNA was performed using GO analysis (http://www.geneontology.org/) based on their molecular function, cellular component, and biological process. Pathway analysis was performed by mapping DE-genes according to the KEGG (http://www.genome.jp/kegg/). A significance threshold of P < 0.05 was applied for both GO and KEGG analysis.

### Coding-non-coding co-expression (CNC) network

The CNC network was constructed to evaluate the correlation between DE-lncRNAs and mRNAs, as described previously^27^. DE-mRNAs associated with the GO enrichments and KEGG pathways were selected, and for coding genes with multiple transcripts, the median value was used. Pearson correlation coefficients (PCC) were calculated for CYP1B1-AS1 with CYP1B1, and for lnc-DKK2 with DKK2. A correlation was considered significant if the absolute PCC was ≥ 0.9 or ≤ −0.9. The CNC network was constructed using Cytoscape (v 3.10.2), and a partial view of the network was manually curated for data representation. In the visualized CNC networks, negative correlations are shown with dashed lines, while positive correlations are indicated by solid lines.

### Polyadenylation assay

Total RNA from THP-1 cells was isolated using Trizol^™^ Reagent and Direct-zol RNA Miniprep Plus kits, followed by 1 h DNase treatment. 1000 ng of RNA was reverse-transcribed with the iScript^™^ Select cDNA Synthesis Kit (Biorad) using oligo-dT primers^23^. The resulting cDNA was used to amplify target genes CYP1B1-AS1, NEAT1 (control lncRNA), CYP1B1, and β-Actin (mRNA control) using PrimeSTAR^®^ HS DNA Polymerase with GC Buffer (Takara Bio) and gene-specific primers (Table S4). PCR products were run on a 2% agarose EtBr gel and imaged using the ChemiDoc Imaging System (Biorad).

### Cellular localization assay

Cytoplasmic and nuclear RNA fractions were separated using the PARIS^™^ kit (Invitrogen^™^). Briefly, 10^7^ THP-1 cells were harvested, re-suspended in the cell fractionation buffer provided, and processed according to the manufacturer’s instructions. A total of 250 ng of RNA from both cytoplasmic and nuclear fractions was reverse-transcribed. The resulting cDNA was used to assess the relative expression of CYP1B1-AS1 in each fraction via qPCR, normalized to ACTB, and compared to the cytoplasmic fraction^23^.

### Protein coding potential assay

Transcripts for CYP1B1-AS1, DANCR (ENSG00000226950), CYP1B1 (ENST00000610745.5), and GAPDH (ENSG00000111640) were synthesized by Integrated DNA Technologies (IDT^™^). To verify the protein-coding potential of CYP1B1-AS1, the transcripts were cloned into a pcDNA-FLAG vector, using Gibson Assembly^®^ Master Mix (NEB) (Table S2). Primer sequences used for construct generation are listed in Table S4. HEK293T cells were seeded at a density of 4 × 10^5^ cells/well in a 24-well plate (Greiner) and transfected with 500 ng of plasmid DNA (Table S2) using Lipofectamine^™^ 3000 (Invitrogen) as per the manufacturer’s instructions. After 24 h of transfection, protein lysates were extracted using M-PER^™^ lysis buffer (Thermo Scientific^™^). FLAG tag signals were detected via western blot using anti-FLAG-tag mouse monoclonal antibody (Invitrogen^™^) to assess the coding potential. Transfection efficiency of constructs was checked prior to western blot and is represented in Fig. S13 (a-d). Additionally, the *in-silico* Coding Potential Assessment Tool (CPAT) (http://lilab.research.bcm.edu/) was employed to further validate the non-coding potential of CYP1B1-AS1 compared to controls.

### Promoter region and TF prediction, and bidirectional promoter dual luciferase assay

The region between CYP1B1-AS1 and CYP1B1, upstream of the +1 site, was screened for active promoter regions, TFBS, and CpG/methylated regions as described previously^42^. A 600 bp promoter region upstream of CYP1B1-AS1 (P_*CYP1B1-AS1*_) was restriction-cloned into the pGL4Luc-Rluc vector (Addgene, #64034) between NheI (NEB) and HindIII (NEB) sites for the dual luciferase assay. The promoter region of ACTB (P_*ACTB*_) was used as a control. Clones and primers used for this assay are listed in Tables S2 and S4 respectively. HEK293T cells were seeded in a 96-well plate (Corning), and 100 ng of plasmid DNA was transfected into each well (n=5) using Lipofectamine^™^ 3000, following the manufacturer’s instructions. Luciferase expression was quantified using Promega kits E2810 for Renilla (R-Luc/RL) and E1500 for firefly luciferase (F-Luc/FL) activity, measured on a BioTek Cytation 5 Reader.

### siRNA mediated knockdown and overexpression of CYP1B1-AS1 and CYP1B1

Knockdown of CYP1B1 and CYP1B1-AS1 in HeLa, THP-1 macrophages, and HEK293T cells was performed using the Lipofectamine^™^ RNAiMAX Transfection Reagent (Invitrogen^™^) according to the manufacturer’s instructions. Silencer^™^ Select Pre-Designed siRNAs s3805 and s3806 (ThermoFisher Scientific) were used to silence CYP1B1(Table S3) (designated as CYPB-KD1 and CYPB-KD2), while siRNAs n507309 and n507310 were used to target CYP1B1-AS1 (designated as lncCYPB-KD1 and lncCYPB-KD2). Silencer^™^ Select Negative Control No. 1 siRNA (ThermoFisher Scientific, #4390844) and GAPDH Positive Control siRNA (ThermoFisher Scientific, #4390850) served as the non-targeting control (NC) and positive control (PC), respectively. Combinations of the siRNAs were used to generate knockdowns; lncCYPB, CYPB, and dCYPB.

To investigate *cis*-regulation of CYP1B1 by CYP1B1-AS1, both genes were silenced in THP-1 macrophages and HeLa cells. Gene expression was assessed by qPCR and western blot. Cells (1 × 10^6^) were seeded in a 6-well plate and transfected with siRNAs (75 pM) using Lipofectamine^™^ RNAiMAX. After 48 h, cells were harvested using Trizol^™^ Reagent (for RNA isolation) and M-PER^™^ lysis buffer (for protein extraction). qPCR analysis quantified CYP1B1-AS1 expression in CYPB-KD1, CYPB-KD2, and CYPB-silenced cells, normalized to HPRT and compared to NC. Protein lysates were analyzed by western blot using anti-CYP1B1 rabbit polyclonal antibody (abcam, #ab185954). Representative whole immunoblots are shown in Fig. S8 (i-l)

For overexpression studies, CYP1B1-AS1 and CYP1B1 were cloned into a pcDNA3.1/Zeo (+) vector (Invitrogen^™^, #V86020) to generate pCYP1B1-AS1 and pCYP1B1 constructs (Table S2) using Gibson Assembly^®^ Master Mix. HEK293T and HeLa cells were seeded in 6-well plates and transfected with 2.5 μg and 1.25 μg of plasmids, respectively, using Lipofectamine^™^ 3000 following the manufacturer’s instructions. Transfection efficiency was monitored by flow cytometry (Fig. S9g). RNA was isolated 24 h post-transfection for qPCR analysis of CYP1B1 expression, normalized to HPRT and compared to untransfected controls.

### mRNA stability assay

To assess the regulation of CYP1B1 by CYP1B1-AS1 at the transcriptional or post-transcriptional level, CYP1B1 mRNA stability was indirectly evaluated by analyzing its half-life as previously described^23, 66^. Briefly, lncCYPB and NC HeLa cells were treated with 5 μg/ml actinomycin D (Gibco^™^). RNA was isolated at 1 h intervals for 5 h post-treatment using Trizol^™^ Reagent, and cDNA was synthesized from 500 ng of RNA for qPCR analysis. The ΔCt of CYP1B1 and HPRT (control) was calculated by subtracting the Ct value at each time point from the value at 0 h (t_0_). Relative mRNA abundance was determined using the 2^(-ΔCt)^ method. The data were then fitted using non-linear regression curve fitting (one-phase decay, ordinary-fit) to calculate the half-lives at 95% confidence intervals (CI).

### RNA isolation, cDNA synthesis and qPCR analysis

For transcript analysis, cells and tissues were harvested at indicated time points in Trizol^™^ Reagent, and RNA was isolated using the Direct-zol RNA Miniprep kit (Zymo Research) with 1-h DNase treatment. 1000 ng of RNA was used for cDNA synthesis (unless otherwise specified) and the synthesized cDNA was diluted 1:20 for each sample. A pool of cDNA from each treated or infected sample was used to generate a 1:10 standard curve, with each standard diluted 1:5 to produce a linear curve. qPCR was performed using SsoAdvanced^™^ Universal SYBR^®^ Green Supermix (Biorad) on a CFX Opus 384 Real-Time PCR System (Biorad). Samples were run in technical triplicates, and the average quantities of test genes were normalized to the endogenous controls ACTB or HPRT, as indicated. Fold expression was calculated by dividing the average of the test sample by the mock or control sample, which was set to 1 unless otherwise specified.

### GE determination for infection and growth curve analysis

For infections, the concentration of axenically grown Cb strains was determined by isolating bacterial DNA using GenElute^™^ Bacterial Genomic DNA Kits (Sigma-Aldrich)^14^. A standard curve was generated from known concentrations of Cb genomic DNA, and absolute quantification of the strains was performed using Taqman probes, *com1* gene-specific primers, and TaqMan^™^ Fast Advanced Master Mix (Applied Biosystems) on a QuantStudio^™^ 6 Flex (Applied Biosystems).

THP-1 macrophages and HEK293T cells were seeded in 24-well plates prior to infection. Cells were treated with siRNA (25 pM) to generate lncCYPB, CYPB, dCYPB, and NC cells. Infections were performed at a MOI of 50 or 100 (as indicated) at 48 h siRNA post-treatment, followed by centrifugation at 500 × g for 10 mins. A second round of siRNA treatment was administered 3 days p.i. to maintain knockdown. Cells were collected on days 0, 1, 4, and 7 p.i., re-suspended in 200 μl of DNA lysis buffer with 10 μl of Proteinase K (20 mg/ml)^14^ DNA was then isolated using the High Pure PCR Template Preparation Kit (Roche), and purified DNA was quantified using qPCR as previously described^14^. GE counts were compared to non-treated cells infected with Cb (NMII) and CbA (Δ*dotA*). For overexpression growth curves, cells were transfected with pCYP1B1-AS1 and pCYP1B1 and infected 24 h post-transfection. Infected cells were collected at indicated time points for GE counts, and qPCR analysis was performed in comparison to controls. All experiments were conducted in technical triplicates and included three biological repeats.

### AHR agonist treatment

THP-1 cells were seeded at a density of 2 × 10^6^ cells/well in a 6-well plate and treated with 100 ng/μl PMA as described previously. THP-1 macrophages were then treated with the AHR agonist FICZ (Sigma-Aldrich) for 1 h. Media containing FICZ was removed and cells were infected with Cb (NMII) at MOI of 50. Infected cells were collected at 24, 48, and 72 h p.i. using Trizol^™^ Reagent for RNA isolation and M-PER^™^ lysis buffer for western blot analysis. CYP1B1-AS1 and AHR expression levels were analyzed by qPCR and compared to the mock control. For western blot, CYP1B1 protein expression in infected cells was compared to controls using an anti-CYP1B1 rabbit polyclonal antibody (Abcam). Densitometry was performed to calculate the fold-expression of CYP1B1, normalized to β-Actin (Abcam) as a loading control.

For growth curve assays, THP-1 macrophages at a density of 2 × 10^5^ cells/well in 24-well plates were treated with FICZ prior to infection. Cells were collected at the indicated time points p.i., and DNA was isolated for GE quantification using qPCR as previously described^14^.

### Indirect immunofluorescence microscopy

THP-1 cells were seeded on coverslips in 24-well plates, treated with siRNA, and infected as described. At 5 days p.i., cells were fixed with 4% paraformaldehyde (PFA) for 20 mins at room temperature. Coverslips were washed with 1X PBS, permeabilized with 0.1% saponin and blocked with 2% BSA + 0.1% saponin. Coverslips were stained with anti-*C. burnetii* guinea pig sera (J. Samuel Laboratory, TAMHSC)^62^, anti-LAMP-1 mouse antibody (Abcam), and Hoechst 33342 (Thermo Scientific^™^) at dilutions of 1:1000, 1:250, and 1:10,000, respectively. Secondary antibodies, anti-guinea pig Alexa Fluor^™^ 647 and goat anti-mouse Alexa Fluor^™^ 488 (InvitrogenTM) were used at 1:1000 dilutions. The stained coverslips were mounted using ProLong^™^ Diamond antifade mountant (Invitrogen^™^) and imaged using an Olympus FV3000 scanning confocal microscope. Quantitative measurements of CCV were performed using ImageJ (NIH) with Qupath plug-in, and Labkit algorithms, with each LAMP-1^+^ bacterial compartment representing an individual CCV.

### Cytokine analysis

THP-1 cells were seeded, treated with siRNA, and infected as described earlier. Supernatants were collected 24 h p.i. from infected and mock controls The supernatants were analyzed by Eve Technologies’ Human Focused 15-Plex Discovery Assay^®^ (Millipore-Sigma) on the Luminex^™^ 200 system, following the manufacturer’s protocol. Briefly, the collected supernatant was centrifuged at 3000 × g for 10 mins at 4°C to remove debris and filter-sterilized twice using Ultrafree-MC sterile filters (Millipore). Cytokine concentrations were measured in biological triplicates, and results were reported as the average of samples in pg/ml.

For mRNA analysis, infected and control cells were collected at 12 h p.i using Trizol^™^ reagent, and RNA was isolated as described earlier. cDNA was synthesized, and qPCR analysis was performed using cytokine-specific primers (Table S4). Gene expression was normalized to HPRT and compared to mock.

### Mice infection with *C. burnetii* NMI

Male and female C57BL/6 mice (6–8 weeks old, n=5) were purchased from Envigo Laboratories and acclimatized in pathogen-free isolator cages with *ad libitum* access to food and water. Experiments were conducted in an Animal-BSL3 (ABSL3) facility in accordance with Texas A&M University’s Institutional Animal Care and Use Committee (IACUC) guidelines and federal regulations. Mice were anesthetized via intraperitoneal injection of ketamine (100 mg/kg) and xylazine (10 mg/kg) for intra-tracheal infection with 10^7^ GE of Cb NMI or PBS (sham) using a Mouse Intubation Platform (Penn-Century^™^) as previously described^12^. Mice were monitored daily for clinical signs and weighed every two days for up to 14 days p.i. At 2, 5, 7, and 14 days p.i., mice were euthanized, and their lungs, spleen, and blood were collected for RNA isolation using Trizol^™^ reagent. Organs were homogenized using a tissue homogenizer (Omni^™^ International), followed by centrifugation at 12,000 × g for 15 mins at 4°C to remove debris. The supernatant was collected, and RNA was isolated as described earlier. Expression of CYP1B1-AS1 and CYP1B1 was quantified by qPCR, normalized to ACTB, and compared to sham-treated controls.

### Flow cytometry analysis

For flow-cytometry based assessment of ROS and apoptosis, cells were seeded at density of 1 × 10^6^ cells/well in 6-well plate, siRNA-treated and infected with Cb at MOI of 50. The assays were performed 48 h post-treatment at indicated time p.i using LSR Fortessa X20 flow cytometer (BD Biosciences).

To measure intracellular ROS, cells were stained with with 20 μM DCFDA/H_2_DCFDA (Abcam) according to the manufacturer’s protocol. Cells were incubated at 37°C in 5% CO_2_ for 30 mins before being re-suspended in 1X buffer (supplied with kit) with 4% FBS. ROS levels were measured at an excitation/emission of 488/535 nm.

For mitochondrial ROS and mass assessment, cells were stained with MitoSOX^™^ Mitochondrial Superoxide Indicators (Invitrogen^™^) and MitoTracker^™^ Green dye (Invitrogen^™^). Briefly, the cells were stained with 5 μM mitoSOX and 200 nM Mitotracker green in 1X PBS and 4% FBS at 37°C, 5% CO_2_ for 15 mins. Mitochondrial ROS and mass were measured at excitation/emission of 510/580 nm and 488/516 nm, respectively.

Mitochondrial membrane potential was assessed using the MitoProbe^™^ TMRM assay (Invitrogen^™^) as per the protocol. Cells were stained with 20 nM TMRM in 1X PBS with 4% FBS for 20 mins at 37°C, 5% CO_2_, and analyzed at 561/585 nm.

For apoptosis assays, FITC-Annexin-V Apoptosis Detection Kit with PI (BioLegend) was used following the manufacturer’s instructions. Cells were stained with 25 nM FITC-Annexin-V in 1X Annexin-V binding buffer, followed by 5 μg/ml PI staining for 5 minutes at room temperature. Annexin-V and PI signals were measured using 488 nm and 610/10 nm lasers, respectively. Gating strategies for the above assays are shown in Fig. S14 (a-d).

### Immunoprecipitation (IP), mass spectrometry and analysis

FLAG-IP- HEK293T cells were seeded at 6 × 10^6^ cells per 10 cm dish and transfected with 14 μg of either pcDNA-CYP1B1 or pcDNA-eGFP (Table S2). For the infection group, one set of pcDNA-CYP1B1 transfected cells at 24 h post-transfection was infected with Cb at MOI of 100. Cells were harvested 48 h post-transfection and lysed in ice-cold RIPA buffer (10 mM Tris-Cl pH 7.5, 150 mM NaCl, 0.5 mM EDTA, 0.1% NP-40, filter-sterilized; Thermo Scientific^™^) supplemented with Halt^™^ Protease and Phosphatase Inhibitor Cocktail (Thermo Scientific^™^). Lysates were centrifuged at 12,000 × g at 4°C for 10 mins, and supernatants were incubated with anti-FLAG M2 beads (Sigma-Aldrich) overnight. IP was performed per manufacturer’s instructions, and samples were eluted using 2× Laemmli Sample Buffer (Biorad), followed by SDS-PAGE on a pre-cast gel (Bio-Rad). Sample bands were excised and sent for LC-MS/MS analysis at UT-Southwestern Proteomics Core.

For CBU0937-FLAG-IP, HEK293T cells were transfected with pCDNA-cGFP or pCDNA-CBU0937 tagged at C-terminal end with a 3X FLAG-tag (Table S2) and IP was carried out as described earlier.

LC-MS/MS- Samples were digested overnight with trypsin (Pierce) after reduction and alkylation with DTT and iodoacetamide (Sigma-Aldrich). Solid-phase extraction was performed using an Oasis HLB plate (Waters). Samples were injected onto an Orbitrap Fusion Lumos mass spectrometer, coupled to an Ultimate 3000 RSLC-Nano liquid chromatography system. Samples were injected onto a loaded onto a 75 μm i.d., 75-cm EasySpray column (Thermo Scientific^™^) and eluted with a gradient from 0–28% buffer B over 90 mins. Buffer A consisted of 2% (v/v) ACN and 0.1% formic acid in water, and buffer B contained 80% (v/v) ACN, 10% (v/v) trifluoroethanol, and 0.1% formic acid in water. The mass spectrometer operated in positive ion mode with a source voltage of 2.5 kV and an ion transfer tube temperature of 300 °C. MS scans were acquired at 120,000 resolution in the Orbitrap, and MS/MS spectra were obtained in the ion trap for 1 s for each full spectrum acquired using higher-energy collisional dissociation (HCD) for ions with charges 2–7. Dynamic exclusion was set for 30 s after an ion was selected for fragmentation.

Spectral data analysis- Raw MS data were analyzed using Proteome Discoverer v 3.0 (Thermo Scientific^™^), with peptide identification through Sequest HT, searching the human reviewed UniProt database. Precursor and fragment tolerances were 10 ppm and 0.6 Da, respectively, with up to three missed cleavages allowed. Carbamidomethylation of Cys was set as a fixed modification, while oxidation of Met was a variable modification. The false discovery rate (FDR) cutoff was set to 1%.

Mass-spec data analysis- Enriched proteins from CYP1B1 IP were compared, with background proteins filtered using the CRAPome 2.0 database. Functional enrichment analysis was performed using GO for molecular function, cellular component, and biological processes. Pathway analysis was performed using the KEGG database, with a significance threshold of P < 0.05. Protein-protein interactions were analyzed using STRING and Cytoscape (v 3.10.2), and a partial network of proteins interacting with CYP1B1 was manually constructed for data representation.

### Western blot and antibodies

For western blot analysis, cell lysates were resolved on 12% pre-cast SDS-PAGE gels (Biorad) and transferred to polyvinylidene difluoride (PVDF) membranes (Millipore-Sigma). The following primary antibodies were used: anti-CYP1B1 rabbit antibody (1:1000; Abcam), anti-FLAG tag mouse monoclonal antibody (1:1000; Invitrogen^™^), anti-β-actin rabbit antibody (1:5000; Abcam), and anti-GAPDH mouse monoclonal antibody (1:3000; Abcam). For detection, the secondary antibodies used were: IRDye^®^ 680RD goat anti-mouse IgG, IRDye^®^ 680RD goat anti-rabbit IgG, and IRDye^®^ 800CW goat anti-rabbit IgG, each at a 1:10,000 dilution. Blots were imaged using a Licor Odyssey Fc imager.

### Statistical analysis

All data are representative of two or more independent experiments (n=3), unless otherwise specified in the figure legends. For all experiments, n refers to the number of biological replicates, wells, or mice. Post-acquisition analysis of flow cytometry data was performed using FlowJo v.10 software (BD Biosciences, USA). Error bars represent the mean ± standard deviation (SD).

Statistical significance was calculated using either a two-tailed unpaired Student’s t-test, one-way ANOVA with Sidak’s post hoc test, or two-way ANOVA with Tukey’s post hoc test. The statistical tests used for each experiment are indicated in the figure legends. All statistical analyses were performed using GraphPad Prism v.10 software (San Diego, USA).

### Key Reagents, Software, and Equipment

All reagents, software, and equipment used in this study, along with their identifiers, are listed in Table S5.

## Data Availability

This paper does not report original code. This study did not generate new unique reagents or strains. All data are available in the main text or the supplementary materials.
